# Prevalence, Antibiogram, and Associated Factors of Bacteria Isolated From Presumptive Meningitis Patients at Debre Markos Comprehensive Specialized Hospital, Northwest Ethiopia

**DOI:** 10.7759/cureus.28500

**Published:** 2022-08-28

**Authors:** Zigale Hibstu, Andargachew Mullu, Adane Mihret, Hylemariam Mihiretie Mengist

**Affiliations:** 1 Medical Laboratory Science, Debre Markos University, Debre Markos, ETH; 2 Virology, Armauer Hansen Research Institute, Addis Ababa, ETH; 3 Immunology, Armauer Hansen Research Institute, Addis Ababa, ETH; 4 Microbiology, Debre Markos University, Debre Markos, ETH

**Keywords:** patients, ethiopia, associated factors, antimicrobial susceptibility, bacterial meningitis

## Abstract

Objective: Bacterial meningitis (BM) is a public health threat with considerable mortality and morbidity worldwide; particularly in the meningitis belt of Africa where Ethiopia is located. The study aims to assess the prevalence, antibiogram, and associated factors of bacteria isolated from presumptive meningitis patients at Debre Markos Comprehensive Specialized Hospital (DMCSH), Northwest Ethiopia.

Methods: We conducted a cross-sectional study between March 1, 2021, and May 30, 2021. Socio-demographic and clinical data were collected using structured questionnaires. Cerebrospinal fluid (CSF) was collected aseptically, and gram stain, culture, and biochemical tests were performed to identify bacterial isolates. An antimicrobial susceptibility test was conducted using the disc diffusion method on Mueller-Hinton agar (MHA). Data were entered into EpiData version 3.1 (Epidata Association, Denmark) and exported to SPSS version 23 software (IBM Corp., Armonk, NY) for analysis. P values ≤ 0.05 at 95% CI were considered statistically significant.

Results: CSF samples from 152 study participants were analyzed and half (50%, 76/152) of them were males. Bacteria were isolated from 17 individuals with an overall prevalence rate of 11.2% (95% CI= 5.9-16.4). The predominant bacterial isolates were *Staphylococcus aureus* (*S. aureus*) and *Klebsiella pneumonia* (*K. pneumoniae) *each accounting for 29.4% (5/17). About 41% (7/17) of the isolated bacteria were found to be multi-drug resistant (MDR) with the predominance of gram-negative bacteria (6/7). Bacteria prevalence was significantly higher in individuals with stiff neck [adjusted odds ratio (AOR), 95% CI, 47.529 (3.2-10.92), P=0.023] and tonsillectomy [AOR, 95% CI, 137.015 (6.25-12.34), P=0.02].

Conclusion: *S. aureus* and *K. pneumoniae* were the leading isolates among presumptive meningitis patients. The alarming presence of a high rate of MDR isolates mandates the need to implement the antibiotic stewardship program in the study setting.

## Introduction

Meningitis is an inflammation of the meninges which is usually acquired spontaneously in the community and/or in hospitals associated with invasive medical procedures [1]. Viruses and bacteria are the most common causes of meningitis which disseminate from the site of infection to the meninges hematogenously [2,3]. Meningitis is the most common and notable infection of the central nervous system (CNS) which can progress rapidly resulting in death or permanent debilitation [4]. Clinically, meningitis is manifested with a combination of fever, headache, meningeal irritation, and altered mental status [5] but adult patients are less sensitive to these symptoms [6]. Its severity may end with deafness, learning disability, visual impairment, and death if not treated timely. Prevalence and etiologies of bacterial meningitis (BM) vary by geography, season, and age of the patient [7].

BM requires quick diagnosis and aggressive therapy [8,9]. It is a life-threatening and public health priority especially in low-income countries [10,11]. According to Colombini et al., a single meningococcal disease in sub-Saharan Africa costs a household 90 USD which is 34% of the annual gross domestic product per capita and it costs 154 USD if it ends with sequelae due to drug resistance and treatment attributed to empirical therapy [12].

As clinical diagnosis alone is not reliable to exclude BM, early isolation of bacteria from cerebrospinal fluid (CSF) before an antimicrobial prescription is highly recommended [10]. Microbiological assessment of the CSF is valuable for successful treatment outcomes and to prevent the increasing rate of antimicrobial resistance [13]. Antimicrobial resistance is a significant issue especially in low-income countries including Ethiopia [14,15] as there is a poor antibiotic stewardship program and self-prescription practice is common.

Meningitis frequently occurs in the meningitis belt including Ethiopia during the dry season i.e., from December to June, and causes frequent epidemics in the country [16]. Different factors are associated with the increased prevalence of BM in the country. BM needs continuous local updates, as etiologic agents have variable nature and resistance to one or more antimicrobials, for effective prevention and control activities. There is a paucity of published data on the bacterial etiologies of meningitis in the study area. This study, therefore, aims to assess the prevalence, antibiogram, and associated factors of bacteria isolated from presumptive meningitis patients at Debre Markos comprehensive specialized hospital (DMCSH), Northwest Ethiopia.

## Materials and methods

Study setting

A hospital-based cross-sectional study was conducted from March 1, 2021, to May 30, 2021, at DMCSH. The Research and Ethical Review Committee of the College of Health Sciences, Debre Markos University issued approval DMU/CHS/RERC/65/2020. Written informed assents (for participants below 18 years old) and consents were obtained from the study participants. The hospital is located in Debre Markos town which is the capital city of East Gojjam zone in Amhara National Regional State, Northwest Ethiopia. The city is located 300 kilometers (KMs) northwest of Addis Ababa, the capital of Ethiopia, and 265 KMs south of Bahir Dar, the capital city of Amhara National Regional State. DMCSH provides health services to over 4 million inhabitants in East Gojjam and the surrounding zones. According to the hospital’s planning department, they provide service for an average of 1,825 patients suspected of meningitis annually.

Inclusion criteria

Presumptive meningitis patients (patients with sudden onset of fever (>38.5°C rectal or >38.0°C axillary) and one or more of the following such as neck stiffness, altered consciousness, another meningeal sign or petechial or purpural rash) were included. However, patients who were on antimicrobial therapy within the prior 14 days and patients with high intracranial pressure were excluded in the study.

Sample size determination and sampling technique

The sample size was determined by using a single population proportion formula considering 95% CI, 5% margin of error, and a 10% prevalence from a previous study conducted in Jimma University Specialized Hospital, Southwest Ethiopia in 2018 [17]. Accordingly, a total of 152 study participants were consecutively enrolled.

Variables

The dependent variable in this study was the prevalence of bacterial isolates. The independent variables include sociodemographic data (sex, age, and residence), vaccination, symptoms, tonsillectomy, history of admission, and presence of comorbidities. 

Data collection

Structured questionnaires and patient record books were used to collect sociodemographic variables and clinical data. Interviewer-administered questionnaires were prepared in Amharic, the local language, to collect data.

Laboratory procedures

Specimen Collection

CSF was collected by experienced physicians under aseptic conditions. A total of 1 mL to 3 mL of CSF specimens were collected by using a sterile syringe from inter lumbar space. Physicians wore gowns, masks, and gloves to avoid contamination while collecting specimens. Because an open and sterile tube was used to collect the fluid, other personnel were to stand away or wear masks to avoid respiratory contamination. Decontamination in an increasing outward fashion with 1%-2% tincture of iodine or chlorhexidine, followed by 70%-90% alcohol was done before skin puncture. After dressing sterile linen over the skin surrounding the puncture site, a needle was inserted, and an adequate volume of CSF was collected in a sterile leakproof tube (1 mL to 3mL). CSF samples were collected in sterile glass tubes for neonates and pediatrics while sterile plastic tubes (5 ml) were used for adults. Then specimens were transported to the hospital laboratory at ambient temperature within 10 minutes and laboratory analyses were started within 30 minutes of specimen delivery.

Isolation and Identification of Bacteria

After collecting CSF specimens aseptically, each specimen was transported to the microbiology laboratory within 10 minutes. All specimens were centrifuged at 30,000 RPM for 15 minutes. Then the supernatant was discarded, and the sediment was re-suspended by pipetting. After performing gram staining, a drop of the suspension was inoculated into blood agar, chocolate agar, and MacConkey agar plates (Oxoid Ltd, Basingstoke, Hampshire, United Kingdom).

MacConkey agar plates were incubated at 370C overnight while blood agar plates and chocolate agar plates were incubated overnight at 370C in a 5% CO2 atmosphere within a candle jar. Positive culture results before 72 hours were further confirmed by biochemical tests and gram staining but culture-negative results were reported after three days. Mannitol salt agar (Oxoid Ltd, Basingstoke, Hampshire, United Kingdom) was used to isolate *Staphylococcus aureus* (*S. aureus*) when beta-hemolysis was observed from the blood agar plate. Gram-positive cocci were identified by biochemical tests, catalase and coagulase positivity, optochin disk sensitivity, bile sensitivity, and bacitracin sensitivity tests. Gram-negative bacteria were identified based on phenotypic characteristics and a series of biochemical tests such as carbohydrate utilization, indole production, mannitol fermentation, citrate utilization, lysine decarboxylation, H2S production, triple sugar iron utilization, and motility testing.

Antimicrobial Susceptibility Testing

The antimicrobial susceptibility screening test was performed using the modified Kirby-Bauer disk-diffusion technique on Muller-Hinton agar (MHA) supplemented with 5% defibrinated sheep blood for fastidious bacterial isolates (Oxoid Ltd) according to the Clinical and Laboratory Standards Institute (CLSI) 2021 guideline [18]. Briefly, 3-5 colonies of the test organism were transferred into a tube containing 3 ml of nutrient broth/normal saline and mixed gently until the suspension becomes turbid and adjusted to 0.5 McFarland standards. Nutrient broth and normal saline were used to standardize the approximate number of bacteria with 0.5 McFarland standards in the Kirby-Bauer disk diffusion method. The suspension was swabbed uniformly onto MHA agar entirely by rotating the plate 60 degrees between streak for non-fastidious organisms, and MHA with defibrinated sterile 5% sheep blood for fastidious organisms. Antimicrobial impregnated disks such as ampicillin (10μg), chloramphenicol (30μg), vancomycin (25μg), imipenem (10μg), oxacillin (30μg), cotrimoxazole (25μg), erythromycin (15μg), penicillin (10μg), azithromycin (15μg), cefotaxime (5μg), cefoxitin (30μg) and cefotaxin (5μg) (Oxoid Ltd, Basingstoke, Hampshire, United Kingdon) were used to assess the antimicrobial susceptibility pattern of gram-positive bacteria. For gram negative bacteria, gentamicin (10μg), amikacin (30μg), trimethoprim-sulphametoxazole (25μg), pencillin G (30μg), meropenem (10μg), chloramphenicol (30μg), erythromycin (10μg), cefotaxin (30μg), cefoxitin (30μg), ampicillin (25μg) and ceftriaxone (30μg) discs were used. They were placed using sterile forceps on the MHA plates’ surface and the plates were incubated (plate placed side up or inverted) at 37°C for 18-24 hours and the zone of inhibition around the disk was measured to the nearest millimeter using a graduated caliper in millimeters. Finally, the isolates were classified as sensitive, intermediate, and resistant to the tested drugs according to CLSI 2021 [18].

Data quality control

A pre-test was performed to assess the quality of the questionnaires before the actual data collection on 5% of the study population in Debre Markos Health center and amendments were made accordingly. The questionnaires were daily checked for completeness. Standard operating procedures were followed during specimen collection, transportation, processing, and interpretation. The quality of culture media and the expiry date of reagents were checked before performing each test. Culture media were prepared aseptically by autoclaving and 5% of each batch was checked for sterility through overnight incubation. The quality and performance of the culture media and antibiotics were also checked by inoculating standard bacterial strains of *S. aureus* (ATCC 25923), *Escherichia coli* (*E. coli*) (ATCC 25922), and *Streptococcus pneumoniae* (*S. pneumoniae*) (ATCC 49619).

Data analysis and interpretations

Data were cleaned, coded, and entered into EpiData version 3.2 software (Epidata Association, Denmark) and exported to SPSS version 23 software (IBM Corp., Armonk, NY) for analysis. Descriptive statistics were used to summarize the data and correct missing variables to minimize errors. Binary logistic regression models were applied to determine the association between predictors and outcome variables with a 95% confidence interval. P values ≤ 0.05 were considered statistically significant.

## Results

Socio-demographic characteristics of the study participants

Half of the study participants were males. Most of the study participants were above 18 years old accounting for 55.26% (84/152) while participants in the age group of 6-18 years were 11.84% (18/152). More than half (51.3%, 78/152) of the study participants were rural residents (Table 1).

**Table 1:** Sociodemographic characteristics of presumptive meningitis patients at DMCSH, Northwest Ethiopia, 2021 *Indicates education starting from kindergarten (zero class), ^$^Indicates unemployed, self-employed, daily laborer, etc. DMCSH - Debre Markos Comprehensive Specialized Hospital

Characteristics		Frequency N (%)	Culture positive N (%)
Sex	Male	76 (50)	10 (13.2)
	Female	76 (50)	7 (9.2)
Age in years	≤5	68 (44.7)	6 (8.8)
	6-18	30 (19.7))	3 (10)
	≥18	54 (35.6)	8(14.8)
Education	Not at all	47(30.9)	8 (17)
	0-8^*^	61 (40.1)	5 (8.2)
	9-12	26 (17.1)	2 (7.7)
	College and above	18 (11.8)	2 (1.1)
Occupation	Farmer	53(34.9)	6 (11.3)
	Merchant	25(16.4)	1 (4)
	Government employee	14(9.2)	1 (7.1)
	Housewife	10(6.6)	0 (0)
	Student	43(28.3)	6 (14)
	Others^$^	7(4.6)	3 (42.9)
Residence	Rural	78 (51.3)	11 (14.1)
	Urban	74 (48.7)	6 (8.1)

Prevalence of bacterial isolates

The overall prevalence of bacterial isolates was 11.2% (17/152) (95% CI= 5.9-16.4). Among culture-confirmed cases, gram-positive bacteria accounted for 59.2% (9/17). The prevalence of BM was 14.1% (11/78) in rural residents and 13.2% (10/76) in males. The highest prevalence of BM was observed among the age group of ≥ 18 years with a rate of 5.26% (8/152) (Table 1). The majority of *Klebsiella pneumonia* (*K. pneumoniae*) 40% (2/5) and *S. aureus* 40% (2/5) were isolated in the age range of 1-10 years.

Among the 17 culture-positive CSF specimens, 82.4% (14/17) showed preliminary gram reaction. We identified seven bacterial species (*S. aureus*, coagulase-negative *Staphylococci* (CoNS), *S. pneumoniae, Listeria monocytogenes, K. pneumoniae, Haemophilus *​​​​*influenzae, *and*Neisseria meningitidis*) in this study. The most predominant bacteria isolated were *K. pneumoniae *and* S. aureus* each accounting for 29.4% (5/17) whereas *Listeria monocytogenes*, CoNS, and *Haemophilus ​​​​influenzae* were the least identified isolates each accounting for only 5.9% (1/17) (Table 2). We did not find multiple isolates from a single specimen.

**Table 2:** Antimicrobial susceptibility pattern of Gram-positive bacterial pathogens among presumptive meningitis patients at DMCSH, Northwest Ethiopia, 2021 AMO=Amoxacillin; AMP=Ampicillin; P=PenicillinG; SXT=Trimethoprim-sulphamethoxazole; CXM=Cefotaxime; CXT=Cefotaxin; FOX= cefoxitin; CEF= Ceftriaxone, C= Chloroamphenicol, IMP=Imipenem, VAN= Vancomtcin, COTRI= CO-trimoxazole, AZI= Azithromycin, OXA= OxacillinS=Susceptible; R= Resistant; NT = Not tested

Bacteria isolated		Antibacterial susceptibility pattern										
		Amp	P	FOX	C	SXT	CXM	IMP	VAN	COTRI	AZITH	OXA
*S. aureus* (n = 5)	S (%)	NT	NT	0	100	NT	NT	NT	NT	NT	NT	NT
	R (%)	NT	NT	100	0	NT	NT	NT	NT	NT	NT	NT
*S. pneumoniae* (n =2)	S (%)	NT	100	100	100	NT	NT	100	100	100	100	NT
	R (%)	NT	0	0	0	NT	NT	0	0	0	NT	NT
CoNS (n= 1)	S (%)	NT	NT	NT	0	NT	NT	NT	NT	NT	NT	100
	R (%)	NT	NT	NT	100	NT	NT	NT	NT	NT	NT	0
*L. monocytogenes* (n = 1)	S (%)	0	NT	0	0	0	0	NT	NT	NT	NT	NT
	R (%)	100	100	100	100	100	100	NT	NT	NT	NT	NT

Antimicrobial susceptibility patterns of bacterial isolates

All *S. aureus* isolates were 100% sensitive to chloramphenicol. We did not find intermediately resistant bacteria (Table 3). All *K. pneumoniae* isolates were 100% susceptible to imipenem, amikacin, and meropenem. However, only one (20%) of the *K. pneumoniae* isolates was susceptible to trimethoprim-sulphamethoxazole. All *K. pneumoniae* isolates were 100% resistant to gentamycin, cefoxitin, ceftriaxone, ampicillin, cefotaxime, and chloramphenicol (Table 3).

**Table 3:** Antimicrobial susceptibility pattern of Gram-negative bacterial pathogens among presumptive meningitis patients at DMCSH, Northwest Ethiopia, 2021 Gen=gentamycin, Fox= cefoxitin, CXM= cefotaxime, Imp= imipenem, Amp= ampicillin, Cxt= cefotaxin, C=chloroamphinecol, Mer=meropenem, P=pencillin, SXT=Trimethoprim-sulphamethoxazole, Cef= Ceftriaxzone; NT = Not tested; S=sensitive; R= resistance

Bacteria isolate	Antibacterial susceptibility test												
		Gen	Fox	Cxm	Imp	Cef	Amp	Amk	SXT	C	Mero	P	Cxt
*K. pneumoniae* (n=5)	S (%)	0	0	0	100	0	0	100	20	0	100	NT	0
	R (%)	100	100	100	0	100	100	0	80	100	0	NT	100
*H. influenzae* (n=1)	S (%)	NT	0	0	NT	100	0	NT	NT	0	NT	0	0
	R (%)	NT	100	100	NT	100	100	NT	NT	100	NT	100	100
*N. meningitidis* (n=2)	S (%)	NT	NT	100	NT	100	NT	NT	NT	NT	NT	0	NT
	R (%)	NT	NT	0	NT	0	NT	NT	NT	NT	NT	100	NT

Multi-drug resistance (MDR) patterns of bacterial isolates 

MDR was reported when bacteria exhibited resistance to at least one agent from two or more classes of drugs. Among the total isolates (n=17), MDR was recorded in 42.2% (7/17) of them. Only one isolate from culture-confirmed gram-positive isolates, six of the gram-negative isolates showed an MDR pattern. *Listeria* *monocytogenes *and* K. pneumoniae* demonstrated an increased level of MDR (Table 4).

**Table 4:** Multidrug resistance patterns bacteria isolated from presumptive meningitis patients at DMCSH, Northwest Ethiopia, 2021

Group	Bacteria	Drugs	Drug class	Level of resistance
Gram-positive	* L. monocytogens* (n=1)	Ampicillin,	Pencillins	100%
		Cefoxitin, cefotaxime, cefotaxin	Third generation cephalosporins	
		Chloramphenicol	Chloramphenicol	
		Co-trimoxazole	Salphonomides	
Gram-negative	*H. influezae *(n=1)	Ampicillin, Pencillin G	Pencillins	100%
		Cefoxitin, Cefotaxime, Cefotaxin, Ceftriaxone	Third generation cephalosporins	
	*K. pneumoniae* (n=5)	Imipenem, Meropenem	Carabapenem	100%
		Gentamycin, Amikacin	Aminoglycosides	
		Trimethoprim sulphamethoxazole	Sulphonamides	
		Cefoxitin, Cefotaxime, Cefotaxin, Ceftriaxone	Third generation cephalosporins	

Factors associated with the presence of bacterial infections

No socio-demographic variables were significantly associated with the presence of BM. Presence of tonsillectomy (p= 0.03) and non-vaccination (p= 0.02) showed significant association with BM in binary logistic regression analysis (Table 5).

**Table 5:** Association of sociodemographic and clinical factors with bacterial isolates among presumptive meningitis patients at DMCSH, Northwest Ethiopia, 2021 *P-value <0.05, OR= Odds ratio; 95% CI=95% confidence interval. NA= Not applicable

Characteristics		Culture positive N (%)	OR (95% CI)	P-value
Sex	Male	10(58.8)	1 1.49(0.54-4.156	0.442
	Female	7(41.2)		
Residence	Rural	11(1 4.1)	1 1.86(0.19-4.54)	0.25
	Urban	6(8.1		
	No	8(9.8)		
Duration of illness	≤a week	14 (15.2)	3.41(0.29-9.74)	0.999
	>a week	57(95)		
Tonsillectomy	Yes	9(17)	13.5.07(1.3-111.5) 1	0.03^*^
	No	8(8.1)		
Vaccination	Yes	6(37.5)	1 6.81(1.3-94.567)	0.02^*^
	No	11(8.1)		
Previous drug use	Yes	6(8.6)	1 0.60(0.578-4.725)	0.349
	No	11(13.4)		
Admission history	Yes	3(15.8)	1 1.59(0.162-2.425)	0.499
	No	14(10.5)		
Comorbidity	Yes	3(15.8)	1.59(0.162-2.425) 1	0.499
	No	14(10.5)		

## Discussion

The overall prevalence of BM in this study (11.2%, 17/152) (95% CI= 5.9-16.4) is consistent with a study conducted at Tikur Anbessa Specialized Teaching Hospital (10.8%) [19], Hawssa University Specialized Hospital (6.9%) [10] and Jimma University Hospital (7%) [17] and other studies from Kenya (11.2%) [20], Iran (11.4%) [21] and India (12.61%) [22], (15.99%) [23]. But it is higher than other studies in Tikur Anbessa Specialized Teaching Hospital (4.7%) [24], Tikur Anbessa and Yekatit 12 specialized hospitals (4.4%) [25], University of Gondar Teaching Hospital (3.8%) [26] and Felege Hiwot Referral Hospital (4.5%) [27] and a study from Palestine (3.9%) [28]. On the contrary, the prevalence of BM in the present study is lower than in a study from Pakistan (25.1%) [29]. These differences could be due to differences in laboratory diagnosis techniques, bacterial endemicity, study population, study design, sampling technique, and geographical location. The higher prevalence of bacteria in this study compared to some studies could be especially since our study was conducted during the meningitis peak season.

Among culture-positive CSF specimens, 52.9% [(9/17), 95% CI=29.4-76.5)] were gram-positive in this study consistent with studies from India (64.71%) [23], (66.18%) [30], Qatar (53%), and Tikur Anbessa and Yekatit 12 Specialized Hospitals (58.8%) 41.2%) [25]. In contrast, the rate of gram-negative bacterial isolates was 47.1% (8/17) (95% CI= 23.5-70.6) in this study which is lower than a study from Palestine (83.6%) [28] and in line with studies from India (35.29%) [23], (64.29%) [22], (28.8%) [30], Qatar (47%), Tikur Anbessa and Yekatit 12 Specialized Hospitals (41.2%) [25] and Felege Hiwot Referral Hospital (62.5%) [27].

Of the isolated bacteria, 41.2% [95% CI= 29.3-57.5] were MDR which is comparable with a study from Dilla University Hospital, Ethiopia 42.1% [8]. *S. pneumoniae* was 100% susceptible to penicillin in the current study but 100% resistant according to a study conducted at Hawassa University Hospital [10]. *K. pneumoniae* showed MDR in this study which is consistent with a study at Dilla University Referral Hospital [8]. The possible reasons for differences in the emergence of resistance may be frequent use of broad-spectrum antimicrobial agents, prolonged use of antimicrobial agents, misuse of drugs, repeated exposure to drugs, and routine empirical treatments. The differences in antimicrobial susceptibility patterns of bacterial isolates against different antimicrobials could be due to differences in bacterial strain, laboratory procedures, bacterial load, laboratory facility, drug control policies, and awareness of the community towards drug resistance.

This study has limitations concerning a short time frame and smaller sample size that results in a small proportion of bacteria compared to other studies. Despite its limitations, our study provides a glimpse into the incidence and antimicrobial susceptibility patterns of culture-confirmed bacterial causes of meningitis important for clinicians and policymakers to design appropriate interventions.

## Conclusions

The prevalence of BM was considerably high in the study area with the predominance of *K. pneumoniae* and *S. aureus*. The presence of tonsillectomy and non-vaccination were significantly associated with the presence of culture-positive bacteria. The isolated bacteria showed either single or MDR patterns. Therefore, strict guidelines and antibiotic stewardship programs should be in place for the prevention and control of antimicrobial resistance. Besides, bacterial isolation and susceptibility testing should be routinely performed before the clinical decision in case of meningitis.
